# Genetic Fusion of an Anti-BclA Single-Domain Antibody with Beta Galactosidase

**DOI:** 10.3390/antib7040036

**Published:** 2018-09-29

**Authors:** George P. Anderson, Lisa C. Shriver-Lake, Scott A. Walper, Lauryn Ashford, Dan Zabetakis, Jinny L. Liu, Joyce C. Breger, P. Audrey Brozozog Lee, Ellen R. Goldman

**Affiliations:** 1 Naval Research Laboratory, Center for Biomolecular Science and Engineering, Washington, DC 20375, USA; george.anderson@nrl.navy.mil (G.P.A.); lisa.shriverlake@nrl.navy.mil (L.C.S.-L.); scott.walper@nrl.navy.mil (S.A.W.); daniel.zabetakis@nrl.navy.mil (D.Z.); jinny.liu@nrl.navy.mil (J.L.L.); joyce.breger@nrl.navy.mil (J.C.B.); 2 The Washington Center for Internships and Academic Seminars, 1333 16th Street N.W., Washington, DC 20036, USA; laurynashford96@gmail.com; 3 Nova Research Inc., Alexandria, VA 22308, USA; plee142@su.edu

**Keywords:** *Bacillus anthracis*, immunoassay, single-domain antibody, genetic fusion, Beta galactosidase

## Abstract

The Bacillus collagen-like protein of anthracis (BclA), found in *Bacillus anthracis* spores, is an attractive target for immunoassays. Previously, using phage display we had selected llama-derived single-domain antibodies that bound to *B. anthracis* spore proteins including BclA. Single-domain antibodies (sdAbs), the recombinantly expressed heavy domains from the unique heavy-chain-only antibodies found in camelids, provide stable and well-expressed binding elements with excellent affinity. In addition, sdAbs offer the important advantage that they can be tailored for specific applications through protein engineering. A fusion of a BclA targeting sdAb with the enzyme Beta galactosidase (β-gal) would enable highly sensitive immunoassays with no need for a secondary reagent. First, we evaluated five anti-BclA sdAbs, including four that had been previously identified but not characterized. Each was tested to determine its binding affinity, melting temperature, producibility, and ability to function as both capture and reporter in sandwich assays for BclA. The sdAb with the best combination of properties was constructed as a fusion with β-gal and shown to enable sensitive detection. This fusion has the potential to be incorporated into highly sensitive assays for the detection of anthrax spores.

## 1. Introduction

*Bacillus anthracis,* the causative agent of anthrax, is a biothreat of grave concern [1,2]. Capable of lethality in both animals and humans, *B. anthracis* has been investigated since the early 1930s for use as a potential bioweapon by several countries around the world. The letter-based attacks of 2001 in the United States is an example of the impact this bacterium has when exploited as a bioweapon. *B. anthracis* spores are easily produced and once aerosolized and disseminated can remain dormant and viable for extended periods. Additionally, cleanup of contaminated areas requires harsh chemical agents and repeated treatments to ensure complete inactivation of the bacterial spores. Much research is centered on developing decontamination methods that are both effective and gentle [3,4], as well as biosensors and reagents for the rapid detection of spores [5,6,7,8]. The Bacillus collagen-like protein of anthracis (BclA), a spore protein, is a good target for antibody development; BclA is an immunodominant glycoprotein and the major component of the hair-like projections that cover the exosporium of *B. anthracis* spores [9,10,11,12].

By nature, antibodies can target and bind to specific antigens. Heavy-chain-only antibodies are found in camelids (camels, llamas, and alpacas) and sharks and lack the light chains that pair with the heavy chains in conventional antibodies [13,14]. Binding takes place through a single unpaired variable heavy domain, which in camelids is known as a VHH. Recombinantly produced VHH are termed single-domain antibodies (sdAbs), or nanobodies [15]. At ~15 kDa sdAbs are about a tenth the size of conventional antibodies; however sdAbs are highly effective in targeting and binding to antigens, while also possessing robust thermal stability and good production characteristics [16,17,18]. Another advantage of sdAbs is that they can readily be engineered and produced as fusions with other protein domains to introduce additional functionalities [19,20,21,22,23,24]. A popular type of fusion is the pairing of a sdAb with the enzyme alkaline phosphatase (AP) [19,20,25,26]. These fusions have two advantages. First, when using a sdAb-AP fusion, there is no need for a secondary antibody, eliminating a step from immunoassays. Secondly, AP is a dimer, so it yields a dimeric binding element with improved apparent affinity due to avidity. The enzyme Beta galactosidase (β-gal) can also be used with secondary reagents in immunoassays [27,28]. Additionally, β-gal is a tetramer with a molecular weight of 464 kDa, so fusions with this enzyme would also benefit from avidity. Previously, it had been reported that the enzyme β-gal is able to function with a scFv (linked variable heavy and variable light chain from a conventional antibody) inserted at the N-terminus of the enzyme [29]. Unlike fusions with AP, the β-gal fusions need to be produced in the cytoplasm. This is because periplasmicly directed β-gal fusions cause lethality which, depending on the fusion, can be due to jamming the translocation pore or misfolding of the β-gal in the periplasm [30,31].

Previously, we described the isolation of sdAbs that recognize several *B. anthracis* spore proteins including BclA [6]. In that work, we constructed a library of phage displayed sdAbs derived from llamas that had been immunized with recombinant spore proteins. Numerous sdAbs that appeared to bind BclA were identified and they fell into several families based on sequence similarity. However, only three of the BclA binding sdAbs were produced and characterized. In the current work, we re-visited the previously isolated BclA binding sdAbs, and characterized four additional clones that had been identified by phage display but neither produced nor tested. These new sdAbs, along with one previously characterized clone, were assessed for their binding kinetics and ability to be integrated into an immunoassay for the detection of BclA. Each was produced in both the periplasm and cytoplasm, and their binding ability and melting temperatures measured. Clone A5, which had been previously characterized, offered the best combination of properties, and thus was further developed as a genetic fusion with β-gal. This sdAb-β-gal fusion was incorporated into an enzyme linked immunosorbent assay (ELISA) for the detection of BclA. We showed that genetic fusions of sdAbs with β-gal provide a route to generate detection reagents.

## 2. Materials and Methods

### 2.1. Reagents

**Figure 1:**
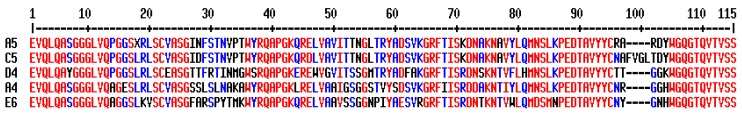
Deduced protein sequences of the five single-domain antibodies (sdAbs) that were evaluated. Sequences have been aligned using Multalin [32] to help identify similarities and differences in the protein sequence of the sdAbs. Red denotes high consensus and blue low consensus. Sequences are given in single letter amino acid code.

The BclA binding sdAbs (A4, A5, C5, D4, E6) had been isolated previously [6]. Sequences are shown in Figure 1.

A C-terminal fragment of BclA (iBclA) was the kind gift of Dr. Michael Weiner (AxioMx, Inc., Branford, CT, USA). The iBclA includes both a His tag as well as a biotinylation tag and was produced and purified as previously described [21]. The iBclA protein is more soluble than non-truncated recombinant BclA and was used for all BclA assays described in this work.

Unless otherwise specified, chemical reagents were from Sigma Aldrich (St. Louis, MO, USA), Thermo Fisher Scientific (Waltham, MA, USA), or VWR International (Radnor, PA, USA). Restriction endonucleases and ligation reagents were from New England Biolabs (Ipswich, MA, USA). DNA amplification was accomplished with the Roche Expand High Fidelity DNA polymerase kit (Sigma Aldrich, St. Louis, MO, USA). Specific kits and assays are defined where applicable.

### 2.2. Periplasmic and Cytoplasmic Production of sdAbs

The coding sequences for the sdAbs were each mobilized from the pecan21 phage display vector into pET22b as NcoI-NotI fragments as described previously [33], or into pET28b using an analogous protocol. The sdAb expression plasmids were transformed into Tuner (DE3) for protein production. Freshly transformed colonies were used to start overnight cultures in 50 mL terrific broth (TB) with appropriate antibiotics (for pET22b: ampicillin 100 µg/mL; for pET28b kanamycin 30 µg/mL) at 25 °C. The next day the overnight cultures were poured into 450 mL of TB with appropriate antibiotics and grown for 2 h at 25 °C prior to induction with isopropyl-β-D-1 thiogalactoside (IPTG, 0.5 mM) and a further 2 h growth.

Purification of sdAbs expressed from pET22b, the periplasmic expression vector, were carried out through an osmotic shock protocol as described previously [34]. For cytoplasmic expression of the sdAbs, purification was similar to the protocol described previously [21] with some minor modifications. Cell pellets were suspended in phosphate-buffered saline (PBS) containing 0.05% Tween 20 prior to sonication, and the immobilized metal affinity chromatography (IMAC) resin was eluted with 0.25 M imidazole prior to purification by fast protein liquid chromatography (FPLC) on a ENrich SEC 70 10 × 300 mm column (Bio-Rad, Hercules, CA, USA). 

### 2.3. Surface Plasmon Resonance

Affinity and kinetics measurements were performed using the ProteOn XPR36 (Bio-Rad, Hercules, CA, USA). Lanes of a general layer compact (GLC) chip were individually coated with BclA and measurements were essentially as described previously [6]. Data analysis was performed with ProteOn Manager 2.1 software, corrected by subtraction of the zero-antibody concentration column as well as interspot correction. The standard error of the fits was less than 10%. Binding constants were determined using the Langmuir model built into the analysis software.

### 2.4. Determining Melting Temperature by Fluorescent Dye Melt Assay

The fluorescent dye melt assay was performed as described previously [35]. Each sdAb was first diluted to a concentration of 500 µg/mL in a final volume of 20 µL PBS. Next a 1:1000 dilution of Sypro Orange dye was added to each sample. Samples were measured in triplicate using a Step One Real-Time polymerase chain reaction (PCR) machine (Applied Biosystems, Foster City, CA, USA). The heating program was run in continuous mode from 25 °C–99 °C at a heating rate of 1% (~2 °C per minute), and data was recorded using the carboxy-X-rhodamine (ROX) filter. The melting point was determined to be the peak of the first derivative of the fluorescence intensity.

### 2.5. Producing Fusion of sdAbs with β-gal

The *E. coli* β-gal was first synthesized and cloned as described previously [36]. Briefly, the β-gal gene from *E. coli* K12 was synthesized by Genscript (Piscataway, NJ, USA) to include a 5’ NotI site and a 3’ XhoI site. The NotI-XhoI fragment was purified and cloned into the pET28b expression vector (Millipore Sigma, Burlington, MA, USA). Next, the sdAb A5 was cloned into the pET28 vector containing β-gal as a NcoI-NotI fragment. Sequencing (Eurofins Genomics, Louisville, KY, USA) confirmed that the constructs were correct.

For protein production, plasmid DNA was transformed to the *E. coli* expression strain BL21(DE3). Colonies were inoculated into 3 mL TB with kanamycin (30 µg/mL) and grown overnight at 25 °C. The next day, 1 mL of the overnight culture was added to 450 mL TB with kanamycin and grown for ~7 h at 30 °C prior to induction (IPTG, 0.5 mM) and then grown overnight at 25 °C. The next morning cells were pelleted and processed as described above for the cytoplasmically grown sdAb.

### 2.6. ELISA

Each sdAb was biotinylated using a 10-fold excess of EZ-Link NHS-LC-LC-Biotin (Thermo Fisher Scientific, Waltham, MA, USA) for 30 min and then the excess biotin was removed using Zeba spin columns. The sdAb concentration was determined by absorbance at 280 nm. ELISAs were performed similar to those described previously [34,37] to determine optimum sdAb pairs. Briefly, wells of 96-well plates (Nunc Maxisorb from Thermo Fisher Scientific, Waltham, MA, USA) were coated by incubating 100 µL capture sdAb (2 µg/mL in PBS) overnight at 4 °C. Wells were washed with PBS containing 0.05% Tween 20 (PBST) and blocked for an hour at room temperature with 4% powdered milk in PBS. After blocking, BclA was added to the wells. For the checkerboard assay 5 µg/mL was used in each well, whereas for dose response, dilution series of BclA were employed. All conditions were measured at least in duplicate, and wells containing just PBS were included as a no-antigen control. After washing with PBST, biotinylated sdAb reporter was added at 2 µg/mL. Wells were washed and then incubated for an hour with streptavidin-horseradish peroxidase (HRP) (100 µL, 1 µg/mL). After a final washing with PBST, signal was developed by the addition of SureBlue (SeraCare, Gaithersburg, MD, USA). Finally, 100 µL 1 N HCl was added to stop color development and the plate read at 450 nm with Tecan Plate Reader (Morrisville, NC, USA).

A dose-response sandwich ELISA was performed with the optimum fusion pair. The plates were coated overnight with 10 µg/mL A5 and blocked for 2 h the next day. Starting with 10 µg/mL β-gal, 1:3 serial dilutions were performed. For the tracer steps, either biotin-labeled A5 (1 µg/mL) followed by streptavidin-HRP and SureBlue or A5-β-gal (1 µg/mL) followed with MuGal (50 µg/mL) to generate a fluorescent product was used. The substrate for HRP is colorimetric and the absorption was read at 450 nm after the color development had been stopped by addition of 1 N HCl. The β-gal assay used a florescent substrate, the fluorescent mixture was excited at 365 nm and the emission collected at 445 nm.

## 3. Results

### 3.1. Evaluating BclA Binding sdAbs 

The BclA binding sdAbs (A4, A5, C5, D4, E6) had been isolated previously [6]; however only A5 from the group had been moved to an expression vector and characterized. In prior work it was determined that A5 could be expressed and produced both in the periplasm as well as in the cytoplasm [6,21,38]. Here, in addition to A5, we chose to revisit anti-BclA sdAbs that had been identified through rounds of phage display panning [6], but were not characterized. Each was cloned into pET22b for periplasmic expression and pET28b for cytoplasmic expression. Table 1 shows representative yields, as well as binding kinetics and melting temperatures for both expression protein variants.

Each of the sdAbs was tested by surface plasmon resonance (SPR) to determine its binding affinity. The on and off rate constants, as well as the calculated dissociation constant (K_D_) from a typical measurement are shown in Table 1. The representative SPR data from which these numbers are derived is shown in Appendix A. Measurements were made at least twice, and generally agreed within a factor of 2 (see Appendix B for a table of average values and the average deviations). The periplasmic version of these sdAbs all had excellent affinity, with low nM or sub nM K_D_ values. Some of the sdAbs, in particular E6, were measured to have much poorer affinity when produced in the cytoplasm.

As an indicator of thermal stability, melting temperatures were measured using a fluorescent-based dye melt assay [39,40]. Results from both periplasmically and cytoplasmically produced sdAbs are reported in Table 1. Three out of the five sdAbs (A5, C5, and D4) showed lower melting temperatures when produced in the cytoplasm.

**Figure 2:**
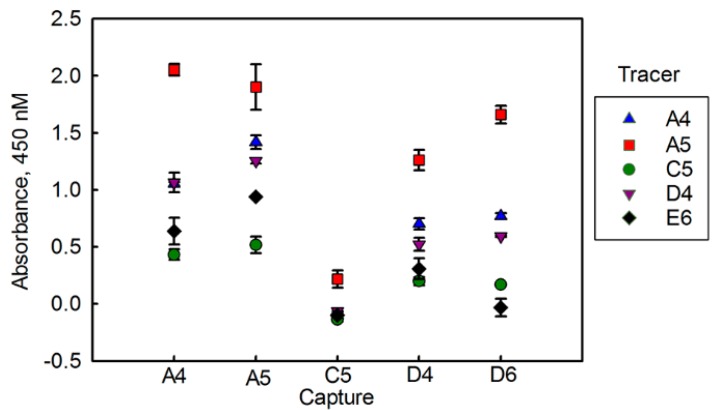
Checkerboard-enzyme linked immunosorbent assay (ELISA) to determine the optimal capture and tracer pairs. Each of the five sdAb captures are shown on the *X* axis and tracers indicated by the symbol (key to the right of the graph). Each tracer sdAb was biotinylated, while the capture sdAbs were adsorbed on the ELISA plate. Measurements were performed in duplicate. The average background (0.41) was subtracted from the average values; error bars represent the average deviation.

**Figure 3:**
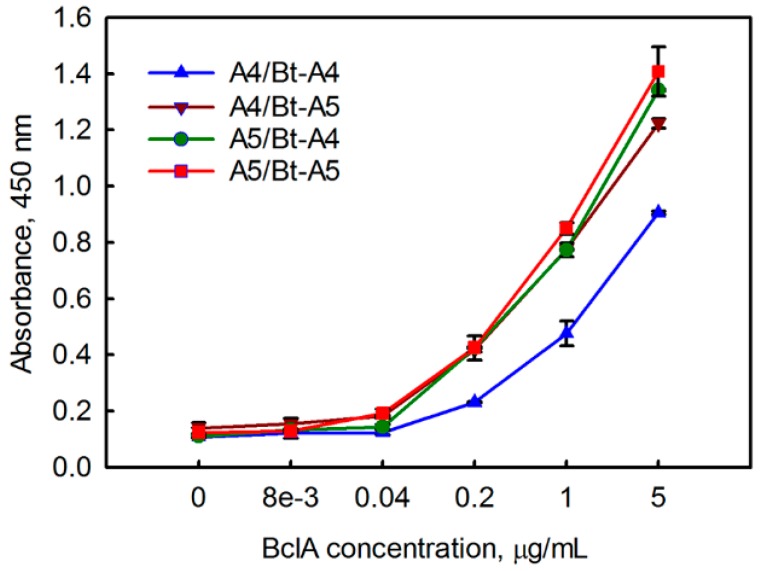
Dose response curves using combinations of A4 and A5 capture/tracer pairs. In each case the tracer is biotinylated (Bt). Measurements were performed in duplicate; the error bars represent the average deviation.

Finally, we evaluated how the five sdAbs perform as both capture and tracer reagents for the detection of BclA in a sandwich format. First, a checkerboard-type assay with a single concentration of BclA was performed (Figure 2). For this experiment, only periplasmically produced sdAbs were examined. Clone C5, with the poorest affinity, also was the worst performer both as a capture and tracer. Overall A4 and A5 performed the best in this assay. An assay using combinations of A4 and A5 capture tracer pairs was performed to determine dose response curves for BclA detection, and to choose the best capture tracer pair (Figure 3). As was seen in the checkerboard assay, the sdAb A5 performed very well as both a capture and tracer, while A4 performed well as a capture or tracer in combination with A5.

From the characterization of the sdAbs, the originally characterized clone, A5, exhibited the best combination of producibility in both the periplasm and cytoplasm, affinity, melting temperature, and function as a reporter in sandwich assays for BclA. Therefore, we chose A5 to produce a fusion with β-gal.

### 3.2. Construction and Evaluation of β-gal Fusion

Before constructing a sdAb fusion, the β-gal gene from *E. coli* was cloned into pET28 for cytoplasmic expression, and the enzymatic function of the construct verified [36]. Once enzymatic functionality had been confirmed, the A5 anti-BclA sdAb was cloned before the N-terminus of β-gal with no linker sequence other than the “AAA” encoded by the NotI restriction site separating the two functional units of the genetic fusion. The fusion construct produced well, with a protein yield of ~25 mg/L.

**Figure 4:**
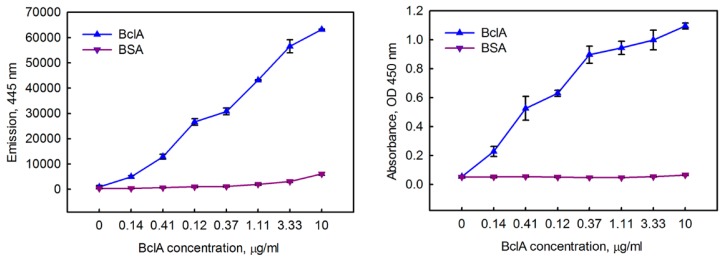
Dose response curves using A5 capture with A5-β-gal tracer (**left**) and A5 capture with Bt-A5 tracer (**right**). Measurements were performed in triplicate; the error bars represent the standard deviation.

The function of the β-gal component of the fusion was demonstrated as previously described [36]. Assessing function of the A5 component was achieved through a sandwich ELISA. The BclA was captured through A5 adsorbed on wells of a 96-well plate. Results are show in Figure 4 in comparison to a standard ELISA using a secondary reagent. Both methods enabled the detection of BclA down to the lowest levels tested (0.14 µg/mL).

## 4. Discussion

Immunoassays based on the detection of spore surface antigen have the potential to be a valuable asset for the detection of *B. anthracis*, the causative agent of anthrax, providing a rapid and facile method to detect spores. Previously, we had isolated several families of sdAbs that recognized BclA [6], a spore protein of *B. anthracis* that has been a target for immunoassays for sensitive spore detection [7] though only a small subset of the isolated sdAbs were initially characterized. One goal of this current work was the construction of a genetic fusion between β-gal and sdAbs. Therefore, it was necessary to examine the ability of each sdAb to be produced and to function when expressed in the cytoplasm. We examined additional anti-BclA sdAbs spanning four additional sequence families, and characterized each in terms of producibility, affinity, melting temperature, and ability to function in immunoassays for BclA. 

As with variable domains from conventional antibodies, VHH contain a pair of cysteines that form an intra-chain disulfide bond. It has been demonstrated that deletion of this conserved disulfide bond, either through mutagenesis or cytoplasmic expression can lead to loss of protein stability, reflected in a lower melting temperature [41,42,43,44]. We observed a lower melting temperature in cytoplasmically produced sdAbs for three out of the five clones examined, suggesting the disulfide fails to form in the reducing environment of the cytoplasm. For clones A4 and E6 the melting temperature measured for cytoplasmically and periplasmically produced sdAb was essentially identical. This may indicate that disulfide bond is either formed or not formed in these two clones regardless of environment. Alternatively, it is possible that the canonical disulfide bond does not contribute significantly to the stability of these particular sdAb clones. Loss of the disulfide bond can also, in some instances, lead to a decrease in affinity for antigen [42]. Among the five BclA binding sdAbs examined, two (D4 and E6) showed a decrease in affinity for BclA when the sdAb was purified from the cytoplasm as determined by SPR. Oddly, E6 was one of the sdAbs for which a decreased melting temperature in the cytoplasmically produced protein was not observed. Perhaps the disulfide bond of E6 contributes more to its affinity than its stability. A definitive assessment of the disulfide bond status in the purified sdAbs could be determined through classical titration methods or a more sensitive mass spectrometry assay to quantify the state of the disulfide bonds in these sdAbs. 

For the majority of the sdAbs examined in this study, we observed sdAb production was compromised when produced in the cytoplasm. In particular, clone A4 decreased from 15 mg/L production in the periplasm to less than 1 mg/mL for cytoplasmic expression. Cytoplasmic expression of A4 was attempted three separate times with low protein expression consistent between the biological replicates. Several researchers reporting high cytoplasmic expression of sdAbs [45] use a specialized *E. coli* strain that promotes disulfide bond formation in the cytoplasm [46,47]. Future work could involve testing expression of sdAb-β-gal genetic fusions in these strains.

After characterizations of their producibility, binding kinetics, and melting temperatures, sdAbs were evaluated for their ability to be employed as either a capture or tracer recognition molecule in an ELISA-type assay. From these studies, A5 functioned well as either capture or tracer reagent; clone A5 also showed good production in the cytoplasm with cytoplasmically produced protein showing the same high affinity as protein produced in the periplasm. This sdAb was then used to form the β-gal fusion. Specific detection of BclA with A5-β-gal fusion as tracer demonstrated the feasibility of using an enzyme-sdAb fusion that can be employed to reduce the number of immunoassay steps and possibly increase sensitivity. 

The availability of β-gal fusions with sdAbs offers an alternative to fusions with AP that could be of great benefit to sensitive detection techniques such as the single-molecule array method [28,48,49,50,51]. Currently these assays require use of secondary reagents; often the sdAb is biotinylated and used in conjunction with streptavidin-β-gal [28,51]. Biotinylation of sdAbs is straightforward; however occasionally it can lead to labeling within the binding loops (complementarity determining regions, CDRs) that participate in antigen binding. In addition, the biotinylated reagent can vary from batch to batch depending on the extent of biotin labeling which can provide inconsistent results. 

## 5. Conclusions

When examining the combination of properties (cytoplasmic expression, affinity, melting temperature, and function in immunoassays for the detection of BclA), clone A5 was the best choice for construction of a β-gal fusion. Previously, researchers demonstrated that β-gal was tolerant to N-terminal fusions and could be produced genetically fused to a scFv for use in immunoassays [29]. We have expanded upon this prior work, demonstrating a functional genetic fusion between β-gal and an sdAb. In general, we have found that sdAb fusion proteins often are more soluble and better produced than fusions with scFv [22]. The fusion of A5 and β-gal expressed well and functioned for the detection of antigen in immunoassays, demonstrating the ability of sdAbs to function in cytoplasmically produced fusions with the enzyme β-gal. Using a sdAb fusion with β-gal eliminates the need to biotinylate the sdAb or to use a secondary antibody, which simplifies the optimization of the assay and reduces assay time. This work demonstrates the feasibility of fusing sdAbs with β-gal for use in detection assays, highlighting the ability to engineer sdAbs. The fusion of A5 with β-gal has the potential to provide sensitive detection of *B. anthracis* spores when integrated into an assay, such as the single-molecule array method, that uses β-gal to generate signal [28,51]. 

## Figures and Tables

**Table 1:** Single-domain antibody (sdAb) properties: yields, binding kinetics, affinity, and melting temperature (Tm).

SdAb	Production	Yield (mg/L)	k_a_ (1/Ms)	k_d_ (1/s)	K_D_ (M)	Tm (°C)
A4	periplasmic	15	9.3 × 10^5^	1.8 × 10^−4^	2.0 × 10^−10^	57
	cytoplasmic	0.3	1.6 × 10^5^	5.8 × 10^−5^	3.7 × 10^−10^	58
A5	periplasmic	7	3.7 × 10^5^	3.7 × 10^−5^	1.0 × 10^−10^	67
	cytoplasmic	6	2.4 × 10^5^	2.8 × 10^−5^	1.1 × 10^−10^	56
C5	periplasmic	6	2.5 × 10^5^	2.7 × 10^−4^	1.1 × 10^−9^	58
	cytoplasmic	4	2.8 × 10^5^	7.5 × 10^−4^	2.7 × 10^−9^	45
D4	periplasmic	11	1.5 × 10^6^	1.9 × 10^−4^	1.3 × 10^−10^	67
	cytoplasmic	3	8.6 × 10^4^	2.1 × 10^−4^	2.5 × 10^−9^	50
E6	periplasmic	20	3.4 × 10^5^	1.2 × 10^−4^	3.6 × 10^−10^	60
	cytoplasmic	5	1.9 × 10^3^	5.7 × 10^−5^	2.9 × 10^−8^	59

## Appendix A

Representative surface plasmon resonance (SPR) data from periplasmically and cytoplasmically produced sdAbs. Concentrations, in nM are noted to the right of each plot.

## Appendix B

Kinetic constants reported as average value/average deviation, based on two measurements.

**SdAb**	**Production**	**k_a_ (1/Ms)**	**k_d_ (1/s)**	**K_D_ (M)**
A4	periplasmic	9.1 × 10^5^/2.2 × 10^4^	1.4 × 10^−4^/4.1 × 10^−5^	1.6 × 10^−10^/4.1 × 10^−11^
	cytoplasmic	1.6 × 10^5^/1.5 × 10^3^	6.6 × 10^−5^/9.5 × 10^−6^	4.3 × 10^−10^/5.6 × 10^−11^
A5	periplasmic	3.6 × 10^5^/9.5 × 10^3^	3.7 × 10^−5^/7.0 × 10^−7^	1.0 × 10^−10^/5.0 × 10^−12^
	cytoplasmic	2.2 × 10^5^/2.2 × 10^4^	2.4 × 10^−5^/3.7 × 10^−6^	1.1 × 10^−10^/6.0 × 10^−12^
C5	periplasmic	2.0 × 10^5^/5.3 × 10^4^	2.2 × 10^−4^/5.3 × 10^−5^	1.1 × 10^−9^/2.5 × 10^−11^
	cytoplasmic	3.0 × 10^5^/2.8 × 10^4^	6.9 × 10^−4^/5.4 × 10^−5^	2.4 × 10^−9^/3.0 × 10^−10^
D4	periplasmic	1.5 × 10^6^/3.0 × 10^4^	2.0 × 10^−4^/1.3 × 10^−5^	1.4 × 10^−10^/1.1 × 10^−11^
	cytoplasmic	9.5 × 10^4^/9.0 × 10^3^	2.3 × 10^−4^/2.1 × 10^−5^	2.5 × 10^−9^/1.0 × 10^−11^
E6	periplasmic	3.0 × 10^5^/3.2 × 10^4^	1.2 × 10^−4^/4.0 × 10^−6^	4.0 × 10^−10^/3.3 × 10^−11^
	cytoplasmic	9.8 × 10^2^/9.4 × 10^2^	4.7 × 10^−5^/9.0 × 10^−6^	6.0 × 10^−7^/5.7 × 10^−7^
